# Resting-state brain network alterations in spinal cord injury patients: an fNIRS study

**DOI:** 10.3389/fnhum.2026.1841779

**Published:** 2026-06-10

**Authors:** Ying Zou, Hyeong Seok Lee, Dae Jung Yang, Yongmei Fan

**Affiliations:** 1 Medical Department, Yueyang Vocational Technical College, Yueyang, Hunan, China; 2 Department of Physical Therapy, Sehan University, Yeongam Gun, Jeollanam Do, Republic of Korea; 3 Department of Rehabilitation, Guilin Hospital of the Second Xiangya Hospital of Central South University, Guilin, Guangxi, China

**Keywords:** functional near-infrared spectroscopy, neuroplasticity, resting-state functional connectivity, sensorimotor function, spinal cord injury

## Abstract

**Objective:**

This study focuses on patients with spinal cord injury (SCI), using healthy subjects as controls, and employs functional near-infrared spectroscopy (fNIRS) to compare the strength of resting-state functional connectivity (rsFC) between SCI patients and healthy subjects, providing neuroimaging evidence for the neuropathological mechanisms of sensory-motor dysfunction and other functional impairments in SCI patients.

**Method:**

Twenty-three healthy adults were included as the health control (HC) group, and 21 SCI patients were included as the SCI group. Data analysis was performed using Nirspark software and SPSS 27.0 to compare the differences in resting-state brain network connectivity between the two groups and to analyze the correlation between brain network connectivity and clinical indicators.

**Results:**

The SCI group showed significantly reduced interhemispheric SMC connectivity at both HbO₂ and HbR levels (*p* < 0.05). HbR-based analysis revealed decreased L-PFC ~ R-M1 connectivity (*p* < 0.01). Interhemispheric SMC connectivity was positively correlated with ASIA sensory and motor scores (*p* < 0.05), and L-PFC ~ R-M1 connectivity was positively correlated with disease duration (*p* < 0.001).

**Conclusion:**

The weakening of interhemispheric SMC connectivity is highly correlated with SCI sensorimotor dysfunction, suggesting it may serve as a potential biomarker for sensorimotor function. Reduced connectivity of PFC-M1 may reflect neural remodeling along common pathways of complex motor function, depression, and neuropathic pain, but this is only a preliminary hypothesis. These quantifiable connectivity features provide potential new targets for monitoring disease progression and evaluating therapeutic interventions, but they need to be further validated in future longitudinal and multimodal studies.

## 1 Introduction

Spinal cord injury (SCI) refers to structural and/or functional damage to the spinal cord caused by traumatic or non-traumatic factors, leading to sensory, motor, and autonomic dysfunction below the level of injury (McDonald and Sadowsky, 2002), severely affecting the patient’s ability to perform activities of daily living (ADL), and bringing a heavy economic and psychological burden to society and families (Levett et al., 2024). Current treatment strategies for SCI, including surgical decompression, neurotrophic agents, and conventional physical rehabilitation, can improve patient function to a certain extent but rarely achieve complete functional recovery (Wenqiang et al., 2025). Therefore, there is an urgent need to find better research and treatment methods to improve the functional deficits of SCI.

Traditional rehabilitation methods usually focus on the recovery of peripheral motor functions rather than directly regulating the central nervous system. However, increasing evidence suggests that neural circuits in the brain undergo functional reorganization after spinal cord injury to compensate for impaired sensorimotor functions (Nardone et al., 2013). This neuroplastic change is considered to play a key role in the recovery process. Despite these advances, there is still a lack of biomarkers validated in large-scale clinical studies to guide the routine treatment of patients with spinal cord injury (Scheuren et al., 2025). Therefore, studying changes in brain function in patients with spinal cord injury and exploring new rehabilitation models through neuroregulation based on biomarkers is expected to bring new hope for the functional recovery of these patients.

To date, studies investigating brain network alterations in individuals with SCI have primarily relied on functional magnetic resonance imaging (fMRI) and electroencephalography (EEG) (Leng et al., 2025; Wang et al., 2024). Although EEG can directly conduct brain electrical signals, it is severely attenuated after passing through the skull, and therefore has the characteristics of high temporal resolution and low spatial resolution (Leng et al., 2025). Although fMRI has high spatial resolution, the scanningenvironment is narrow and noisy, and it requires patients to remain completely still. These characteristics may cause significant discomfort and excessive motion artifacts in patients with radiculopathy or spinal instability (Wang et al., 2024).

Functional near-infrared spectroscopy (fNIRS) provides a feasible alternative. As a non-invasive optical neuroimaging technique, fNIRS measures cortical hemodynamic activity by detecting changes in the absorption of near-infrared light by oxygenated and deoxygenated hemoglobin (neurovascular coupling response) (Zhang et al., 2025; Chen X. et al., 2025). fNIRS has several notable advantages: good compromise, achieving a better balance among temporal and spatial resolution, cost, and portability; high tolerance to subject movement; silent operation, minimizing patient anxiety and discomfort; and its portability allows patients to be assessed in a more friendly environment (Scarapicchia et al., 2017). Based on these advantages, we believe that fNIRS is more suitable for capturing changes in the brain networks of SCI patients. To our knowledge, there is currently a lack of resting-state brain network studies of SCI patients based on fNIRS. Whole-brain network analysis is a commonly used mainstream analysis paradigm (Chen R. et al., 2025). However, multiple resting-state fMRI studies have confirmed that SCI patients exhibit a significant reduction in functional connectivity of the sensorimotor cortex (Oni-Orisan et al., 2016; Pan et al., 2017). Moreover, motor and sensory impairments are associated with different patterns of brain reorganization, which, although involving the visuospatial network, primarily center on the sensorimotor cortex and related cortical regions (Brihmat et al., 2026). Therefore, focusing on the sensorimotor cortex and prefrontal cortex can serve as the main targets for capturing post-SCI brain functional reorganization. Analyzing specific brain regions at the same time can effectively avoid the multiple comparison problem associated with whole-brain analysis and improve the statistical power of studies with medium to small sample sizes, so targeted analysis of these areas can more sensitively detect neuroplastic changes related to sensory-motor functions. This study hypothesizes that the resting-state brain networks of healthy controls and the SCI group will differ, with SCI patients showing reduced connectivity in the sensorimotor network, and analyzes the correlation between regional brain network connectivity strength and functional impairment assessment metrics. We expect that changes in sensorimotor network connectivity will be associated with sensorimotor dysfunction (measured by ASIA sensory and motor scores), while changes in prefrontal-sensorimotor cortex connectivity may be related to more widespread functional disability.

## 2 Materials and methods

### 2.1 Participants

This study was conducted with 21 patients with spinal cord injury who were hospitalized in the Department of Rehabilitation Medicine at the Second Xiangya Hospital of Central South University, Changsha, China, and 23 healthy adults. The patients with SCI were assigned to the SCI group, while the healthy adults were assigned to the health control (HC)group. Demographic information, including sex, age, education level, and the Edinburgh Handedness Inventory, was collected.

The inclusion criteria for the SCI group were as follows: (1) classification as grade A, B, C, or D according to the revised International Standards for Neurological Classification of Spinal Cord Injury by the American Spinal Injury Association (Kirshblum et al., 2011), (2) age between 40 and 70 years and at least 6 years of formal education, (3) disease duration of at least 1 month, (4) right-handedness, (5) presence of lower-extremity motor dysfunction after SCI, (6) intact consciousness without dementia or aphasia and the ability to follow instructions, (7) no history of psychiatric disorders, epilepsy, stroke, or other neurological diseases, (8) absence of severe internal diseases, such as cardiac, hepatic, pulmonary, or renal disorders, and the ability to participate in the study as an inpatient. The exclusion criteria were: (1) skull defects or a history of cranioplasty involving metallic materials, (2) concomitant traumatic brain injury or lower-extremity peripheral nerve injury, (3) use of any medications known to significantly affect resting-state brain network functional connectivity within 4 weeks prior to enrolment and throughout the study, including but not limited to baclofen, gabapentin, pregabalin, opioids, or antidepressants (fluoxetine within 8 weeks prior to enrolment). The general characteristics of the SCI group are presented in Table 1.

**Table 1:** Spinal cord injury patient data.

No.	Level of injury	Disease duration (months)	ASIA Impairment scale	Motor score	Sensory score
1	T11	10	B	50	184
2	T10	1	B	50	180
3	T7	2	A	50	112
4	T5	12	B	50	160
5	T12	1	C	64	188
6	C4	1	D	40	124
7	C4	2	D	42	124
8	T7	10	D	70	168
9	C4	11	B	0	124
10	C5	9	C	18	128
11	C5	1	D	44	128
12	L1	6	D	76	192
13	T11	2	C	62	184
14	C7	12	D	72	184
15	T6	3	C	60	164
16	T6	1	B	50	164
17	C3	9	B	0	120
18	L1	24	D	84	192
19	C4	5	C	16	124
20	T12	3	C	66	188
21	C2	4	A	0	8

ASIA, American Spinal Injury Association.

The inclusion criteria for the HC group were: (1) age between 40 and 70 years with at least 6 years of education, (2) right-handedness, (3) absence of mental or physical disorders and ability to participate in the study based on physical examination results. The exclusion criteria were: (1) age younger than 40 years or older than 70 years, (2) current or previous severe organic or functional disorders, (3) inability to comply with study procedures.

All participants and their caregivers were informed about the study procedures and purpose, and written informed consent was obtained before participation. This study was approved by the Ethics Committee of the Second Xiangya Hospital of Central South University, Changsha, China (approval number: LYG2021062).

### 2.2 Resting-state recording protocol

fNIRS data were collected under resting-state conditions. The total resting period was set to 10 min. To allow participants to adapt to the experimental environment and to ensure that the fNIRS signals reached a stable state, an additional 10 s of baseline resting data were recorded prior to the measurement, resulting in a total recording duration of 610 s. During the experiment, participants wore an optical probe headgear equipped with the fNIRS system and were placed in a quiet room in a comfortable supine position. They were instructed to keep their eyes open, remain still, and refrain from performing any specific cognitive or motor tasks throughout the recording period.

### 2.3 Data Collection

#### 2.3.1 ASIA sensory and motor score

Neurological function was evaluated by a certified physician using the International Standards for Neurological Classification of Spinal Cord Injury (ISNCSCI). The motor score was assessed in 10 pairs of key muscle groups in the upper and lower extremities, each graded on a 0–5 scale, yielding a maximum total score of 100 points. The sensory score was evaluated at 28 dermatomes on each side for light touch and pinprick sensation, each graded on a 0–2 scale, resulting in a maximum total score of 224 points.

#### 2.3.2 fNIRS data acquisition

**Figure 1:**
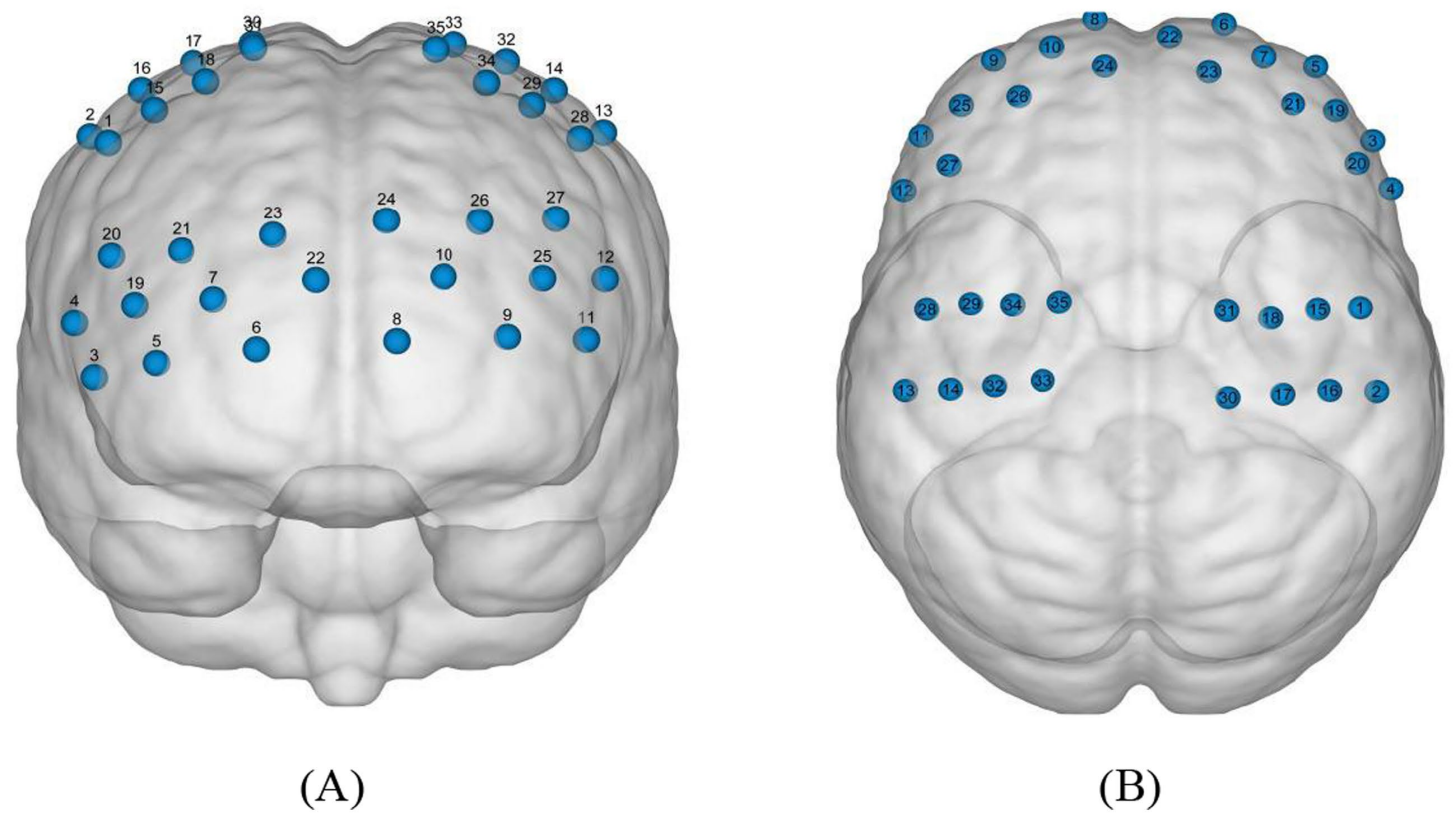
Channel arrangement. **(A)** Is the front view; **(B)** is the top view. The blue dot represents the passage.

Cerebral hemodynamic responses were measured using an fNIRS system (NirSmart II-3000B, Danyang Huichuang Medical Equipment Co., Ltd., China). The system employed two wavelengths, 730 nm and 850 nm, with a sampling rate of 11 Hz. A custom-designed cap equipped with 14 light sources and 14 detectors was used, forming a total of 35 valid channels covering the prefrontal and sensorimotor cortices. The channel configuration is illustrated in Figure 1.

The center of each channel was defined as the reference point representing the primary cortical region detected by that channel. Based on anatomical locations and corresponding Brodmann areas, four regions of interest (ROI) were defined: the left sensorimotor cortex (L-SMC), right sensorimotor cortex (R-SMC), left prefrontal cortex (L-PFC), and right prefrontal cortex (R-PFC). The channel distribution for each ROI is shown in Table 2.

**Table 2:** Condition according to ROI channel classification.

ROI	Number of channels	Corresponding channels
L-SMC	8	13/14/28/29/32/33/34/35
R-SMC	8	1/2/15/16/17/18/30/31
L-PFC	9	8/9/10/11/12/24/25/26/27
R-PFC	9	3/4/5/6/7/19/20/21/23

ROI, Region of Interest; L-SMC, Left Sensorimotor Cortex; R-SMC, Right Sensorimotor Cortex; L-PFC, Left Prefrontal Cortex; R-PFC, Right Prefrontal Cortex.

### 2.4 fNIRS data analysis

#### 2.4.1 Signal processing

The collected raw fNIRS data were preprocessed using Homer3. (1) Data trimming: Remove the first 10 s of data to exclude initial unstable signals; (2) Signal quality assessment: Calculate the coefficient of variation (CV); for each channel, a CV value>20% is considered a poor channel. If the number of bad channels was ≥ 30% for a participant, the participant’s data were deemed unacceptable and excluded from further analysis; (3) Optical density conversion: Convert the raw data to optical density; (4) Motion artifact correction: Correct motion artifacts using a spline interpolation algorithm; (5) Band-pass filtering: Apply a 0.01–0.1 Hz band-pass filter to remove slow drift and high-frequency physiological noise (such as heartbeats and respiratory cycles) (Scarapicchia et al., 2017); (6) Physiological noise removal: Spatial filtering using principal component analysis, retaining the principal components that cumulatively explain 80% of the total variance; (7) Conversion to hemoglobin concentration: Calculate differential pathlength factor (DPF) values for two wavelengths and convert optical density to relative concentrations of HbO2/HbR/HbT using the modified Beer–Lambert law (Nguyen et al., 2018).

#### 2.4.2 Functional connectivity analysis

For each participant and each hemoglobin type (HbO2, HbR, HbT), a 35 × 35 functional connectivity (FC) matrix was generated. FC values are obtained by calculating the Pearson correlation coefficient r between all possible channels in 600-s resting-state segments during the preprocessed time series, and using the Fisher-Z transformation to make the data closer to a normal distribution. This process produced a set of three symmetric matrices for each subject, representing the functional connectivity strength across the entire measurement area.

### 2.5 Statistical analysis

Demographic variables were analyzed using IBM SPSS 27.0 and compared via independent t-tests or chi-square tests. ROI FC and ASIA scores were analyzed using a three-step strategy: Step 1 (between-group differences): Independent sample t-tests were used to compare ROIs FC in SCI patients and healthy controls, with False Discovery Rate (FDR) correction applied. A *p*-value < 0.05 indicated a significant difference, with effect size calculated using Cohen‘s *d*. This step was completed using NirSpark software (version 1.8.9). Step 2 (raw correlations): Within the patient group (n = 21), the normality of functional connectivity strength in differing brain regions and ASIA scores was verified. If normal, Pearson correlation analysis was used to assess their relationship, with Spearman analysis performed for sensitivity testing. Step 3 (adjusted correlations): To further control for potential confounding factors (disease duration, ASIA grade, injury level), partial correlation analysis was conducted within the patient group to calculate the net correlation coefficient (partial *r*) after removing confounders. Steps two and three analyses were carried out using SPSS 27.0, with significance set at *p* < 0.05.

## 3 Results

### 3.1 Comparison of demographic data

Demographic data were evenly distributed between the two groups (all *p* values > 0.05), see Table 3.

**Table 3:** Demographic characteristics.

Variables	HC group (*n* = 23)	SCI group (*n* = 21)	*p*
Age (years)	51.78 ± 8.11	50.86 ± 7.05	0.69
Gender (male/female)	14/9	14/7	0.69
Education (years)	11.96 ± 2.16	11.71 ± 3.13	0.77
EHI (%)	84.1 ± 5.9	84.3 ± 7.3	0.94

Values are Means ± Standard (SD). EHI, Edinburgh Handedness Inventor.

### 3.2 Changes in functional connectivity

#### 3.2.1 Brain functional connectivity based on HbO₂ levels

**Figure 2:**
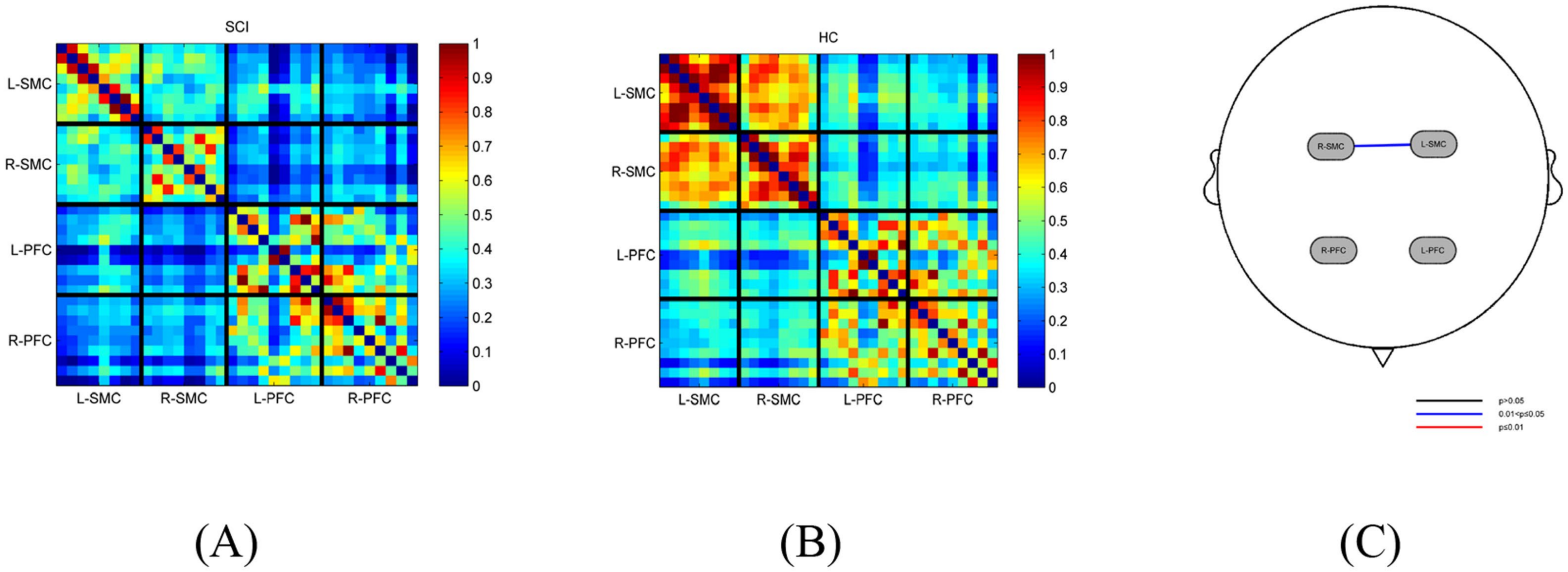
Comparison of functional connectivity strength between ROIs in the two groups at the HbO2 level. **(A)** Functional connectivity strength matrix between ROIs in the SCI group at the HbO2 level. **(B)** Functional connectivity strength matrix between ROIs in the HC group at the HbO2 level. **(C)** ROI connections showing significant differences between the SCI and HC groups among 4 ROIs at the HbO2 level.

In the SCI group, the R-SMC ~ L-SMC FC was significantly lower than that in the HC group [SCI: 0.39222 ± 0.21654 vs. HC: 0.64925 ± 0.30485, *t* (44) = 3.19, FDR-corrected *p* < 0.05, Cohen‘s d = 0.96, see Figure 2].

#### 3.2.2 Brain functional connectivity based on HbR levels

**Figure 3:**
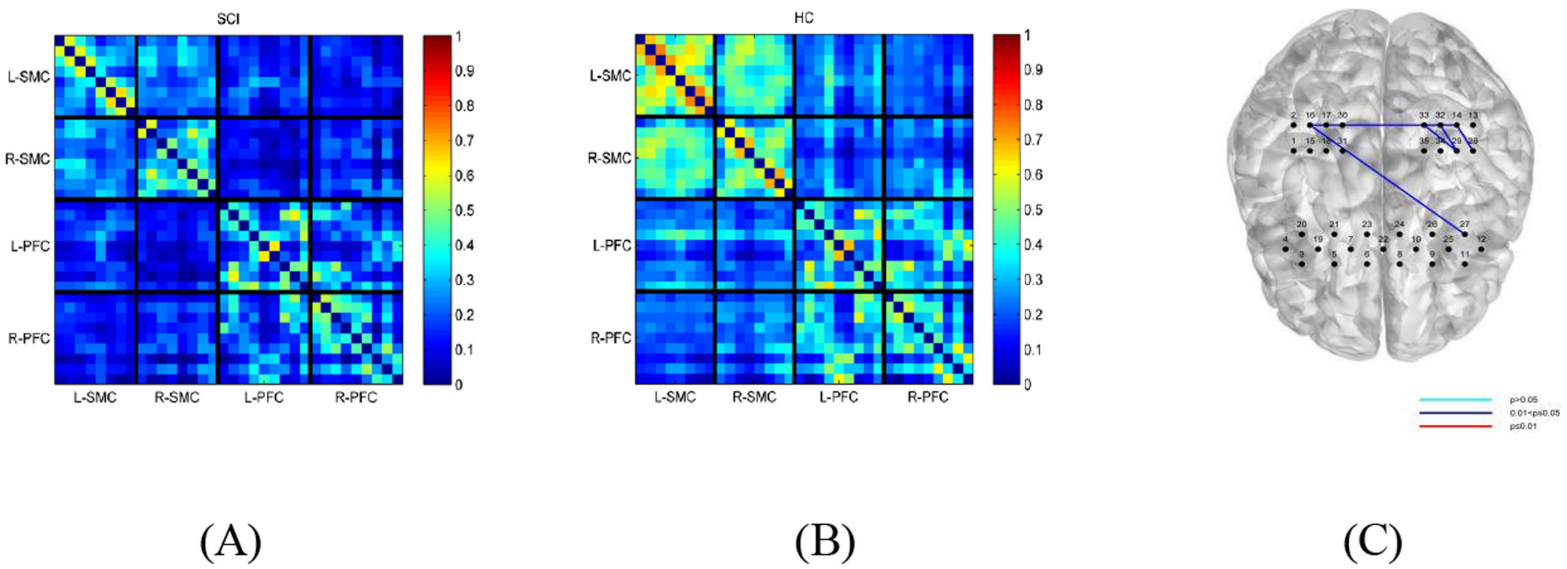
Comparison of functional connectivity strength between the two groups at the HbR level. **(A)** Functional connectivity strength matrix between ROIs in the SCI group at the HbR level. **(B)** Functional connectivity strength matrix between ROIs in the HC group at the HbR level. **(C)** Display of the functional connectivity of ROIs with significant differences between two groups at the HbR level on a channel-level topology map.

In the SCI group, the R-SMC ~ L-SMC FC and the L-PFC ~ R-SMC FC were significantly weaker than those in the HC group [R-SMC ~ L-SMC: HC: 0.47428 ± 0.24777 vs. SCI: 0.27646 ± 0.21956, *t* (44) = 2.79, FDR-corrected *p* < 0.05, Cohen‘s *d* = 0.84; L-PFC ~ R-SMC: HC: 0.23165 ± 0.12488 vs. SCI: 0.11114 ± 0.12903, *t* (44) = 3.14, FDR-corrected *p* < 0.05, Cohen‘s *d* = 0.95，see Figure 3]. The reduction in connectivity was most pronounced between the L-PFC and the right primary motor cortex (R-M1) [HC: 0.41839 ± 0.249395 vs. SCI: 0.072056 ± 0.25928, *t* (44) = 4.51, FDR-corrected *p* < 0.05, Cohen‘s *d* = 1.36, see Figure 3].

### 3.3 Clinical relevance

**Figure 4:**
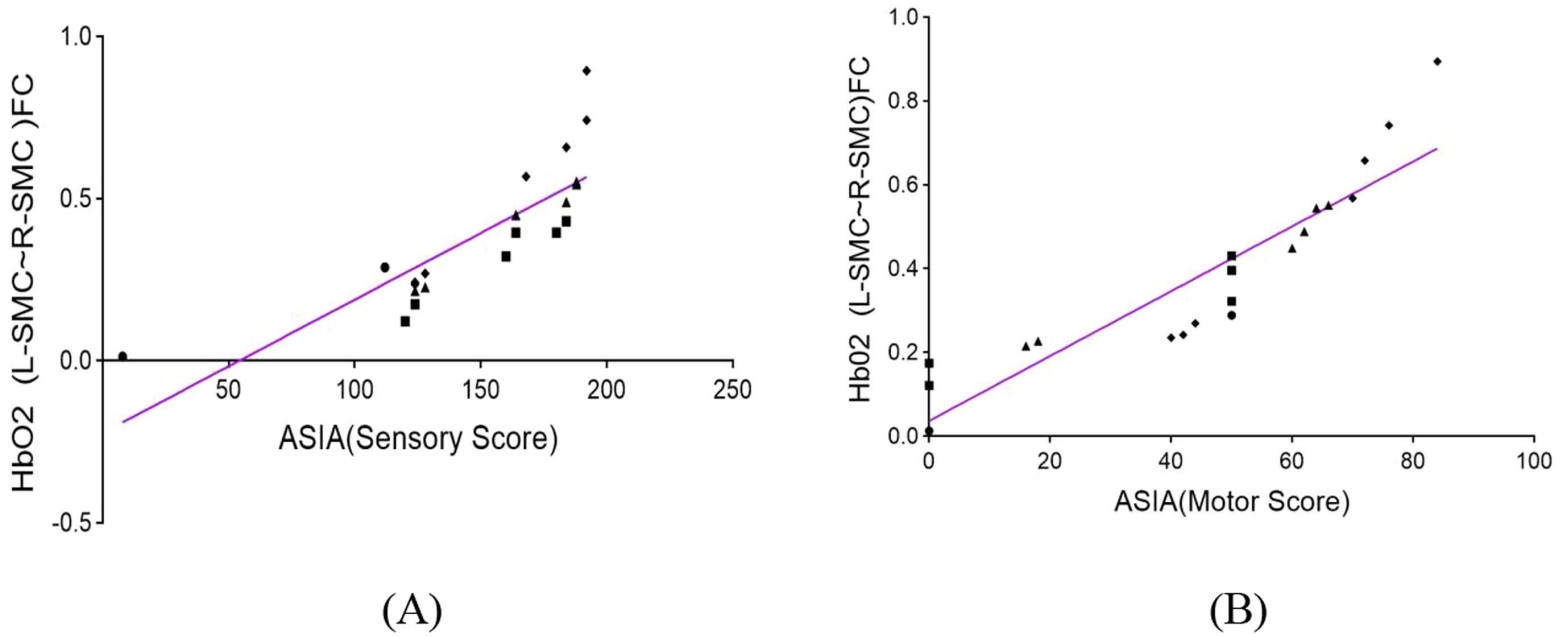
Correlation between the L-SMC~R-SMC connection strength at HbO2 level and ASIA sensory and motor scores. **(A)** Scatter plot of the correlation between the connection strength of L-SMC~R-SMC and ASIA sensory scores in the SCI group. **(B)** Scatter plot of the correlation between the connection strength of L-SMC~R-SMC and ASIA motor scores in the SCI group. The dots represent ASIA A, the squares represent ASIA B, the triangles represent ASIA C, the diamonds represent ASIA D.

Figure 4 shows that at the HbO2 level, Pearson correlation analysis indicated that the L-SMC ~ R-SMC FC was positively correlated with both the ASIA sensory score (*r* = 0.823, *p* < 0.001) and the ASIA motor score (*r* = 0.908, *p* < 0.001). Spearman sensitivity analysis showed that the FC was also positively correlated with the ASIA sensory score (*r*_s_ = 0.911, *p* < 0.001) and the ASIA motor score (*r*_s_ = 0.922, *p* < 0.001). After controlling for disease duration, lesion plane, and ASIA grading as confounding factors, partial correlation analysis showed that the L-SMC ~ R-SMC FC remained positively correlated with the ASIA sensory score [partial *r*(16) = 0.531, *p* = 0.023] and the ASIA motor score [partial *r*(16) = 0.748, *p* < 0.001], supporting the robustness of the main results.

**Figure 5:**
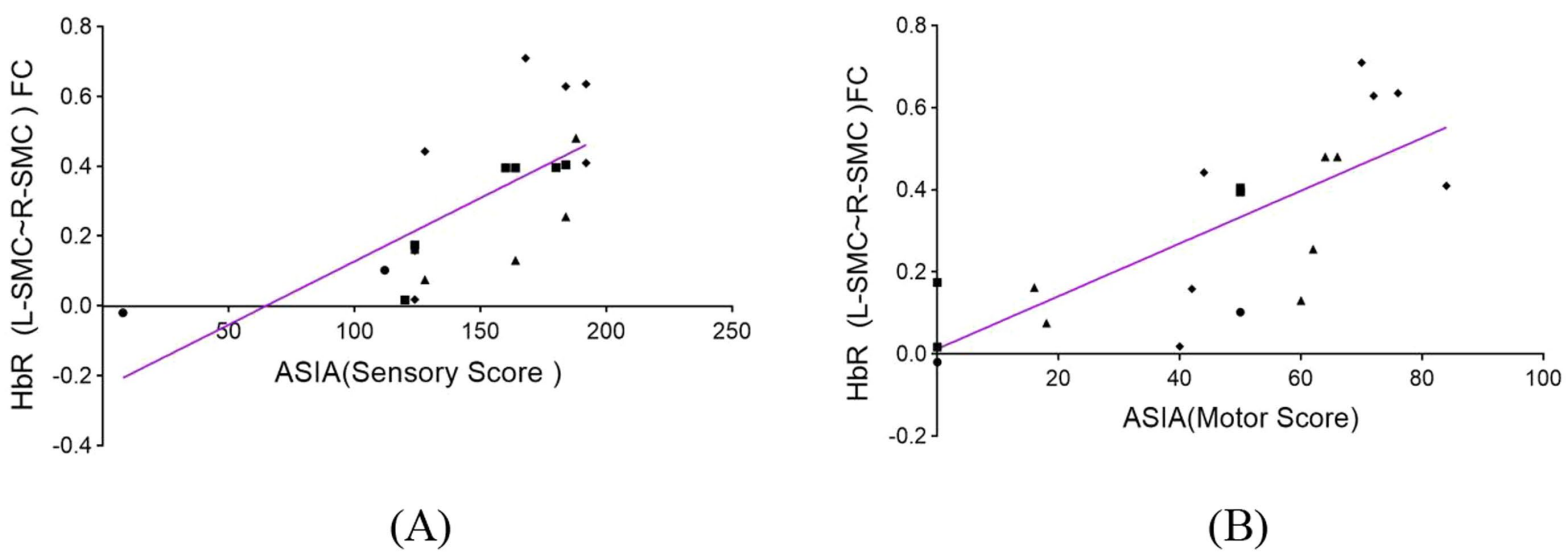
Correlation between the L-SMC~R-SMC connection strength at HbR level and ASIA sensory and motor scores. **(A)** Scatter plot of the correlation between connection strength of L-SMC~R-SMC and ASIA sensory scores in the SCI group. **(B)** Scatter plot of the correlation between connection strength of L-SMC~R-SMC and ASIA motor scores in the SCI group. The dots represent ASIA A, the squares represent ASIA B, the triangles represent ASIA C, the diamonds represent ASIA D.

Figure 5 shows that, at the HbR level, Pearson correlation analysis indicated that the L-SMC ~ R-SMC FC was positively correlated with both the ASIA sensory score (*r* = 0.719, *p* < 0.001) and the ASIA motor score (*r* = 0.746, *p* < 0.001). Spearman sensitivity analysis showed that the FC was also positively correlated with the ASIA sensory score (*r*_s_ = 0.809, *p* < 0.001) and the ASIA motor score (*r*_s_ = 0.785, *p* < 0.001). After controlling for disease duration, lesion plane, and ASIA grading as confounding factors, partial correlation analysis showed that the L-SMC ~ R-SMC FC remained positively correlated with the ASIA sensory score [partial *r*(16) = 0.478, *p* = 0.045] and the ASIA motor score [partial *r*(16) = 0.562, *p* = 0.015], supporting the robustness of the main results.

**Figure 6:**
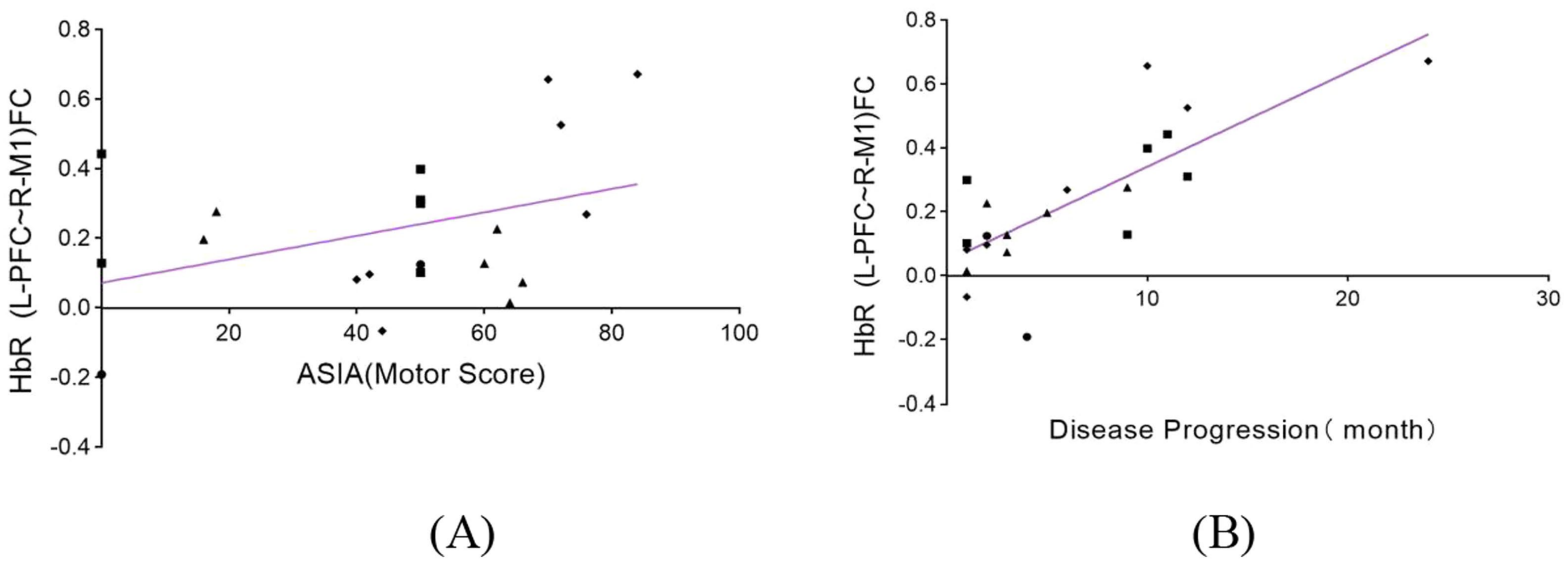
The correlation between the L-PFC~ R-M1 connectivity strength at the HbR level and AISIA motor scores and disease course. **(A)** Scatter plot of the correlation between L-PFC~R-M1 connectivity strength and ASIA motor scores in the SCI group. **(B)** Scatter plot of the correlation between L-PFC~R-M1 connectivity strength and disease duration in the SCI group. The dots represent ASIA A, the squares represent ASIA B, the triangles represent ASIA C, the diamonds represent ASIA D.

Figure 6 showed that at the HbR level, Pearson correlation analysis indicated that the L-PFC ~ R-M1 FC was not significantly correlated with ASIA motor scores (*r* = 0.388, *p* = 0.082), but was positively correlated with disease duration (*r* = 0.772, *p* < 0.001). Spearman rank correlation analyses confirmed these patterns: ASIA motor scores (*r*_s_ = 0.335, *p* = 0.138) and disease duration (*r*_s_ = 0.758, *p* < 0.001). After controlling for injury level and ASIA grade, partial correlation analysis showed that the connectivity strength remained not significantly correlated with ASIA motor scores [partial *r*(17) = 0.338, *p* = 0.157], while remaining positively correlated with disease duration [partial *r*(17) = 0.798, *p* < 0.001], supporting the robustness of the main results.

## 4 Discussion

This study provides evidence of characteristic changes in brain networks of SCI patients based on fNIRS. Specifically, we observed that interhemispheric SMC functional connectivity was significantly reduced at both HbO2 and HbR levels. In addition, HbR analysis showed weakened connectivity between the L-PFC and R-SMC, which was particularly evident in the connection between L-PFC and R-M1. Notably, the interhemispheric SMC connectivity strength at the HbO2 and HbR level was significantly positively correlated with ASIA sensory and motor scores, and the connectivity strength between L-PFC and R-M1 at the HbR level was positively correlated with disease duration.

In fNIRS, the concentration of HbO2 represents the local cerebral oxygen supply state; the concentration of HbR reflects the local oxygen extraction fraction of the brain (Zhang et al., 2025) and has a lagged response. Therefore, high connectivity in brain networks analyzed by HbR represents tighter neurovascular coupling. Both HbO2 and HbR reflect local cerebral blood flow and metabolism, with correlation coefficients ranging from 0.62 to 0.99, indicating a strong correlation between the two (Wang et al., 2011). Therefore, this study conducted brain connectivity analysis at both HbO2 and HbR levels.

Interhemispheric SMC connectivity was reduced at both the HbO2 and HbR levels. Although there was some clinical heterogeneity among individuals with SCI in this study, increasing neuroimaging evidence suggests that patients with different disease courses, injury levels, and ASIA impairment grades exhibit an overall trend of reduced functional connectivity within the sensorimotor network during resting state (Sun et al., 2022; Brihmat et al., 2026; Vallesi et al., 2022). This phenomenon is consistent with the results of this study, indicating that despite clinical heterogeneity, the sensory and motor deficits caused by deafferentation and de-efferentation due to spinal cord injury may drive a common trend in brain functional reorganization. This mechanism aligns with the principle found in developmental studies of ‘hemispheric coordination depending on shared sensorimotor experience—passive decoupling’ (Wu et al., 2025). Notably, studies have shown that after moderate to severe stroke, interhemispheric connectivity is actively weakened, accompanied by enhanced connectivity between the unaffected premotor cortex and subcortical structures, which compensates for the function of the affected side (Fan et al., 2026). Although the current study only assessed interhemispheric connectivity, findings from stroke research provide a complementary perspective—reduced interhemispheric coupling is often accompanied by enhanced ipsilateral compensatory connections. In this study, right-handed patients with SCI showed weakened connectivity in the interhemispheric SMC. Drawing on the mechanisms of ‘developmental asymmetry in the brain’ and ‘asymmetric compensation’ observed in stroke research, we speculate that, against the backdrop of reduced interhemispheric interaction, these patients’ brains may exhibit enhanced lateralized connections between the dominant hemisphere and remaining pathways to support motor function recovery. This framework shifts the interpretation of interrupted interhemispheric connections from purely pathological reorganization to context-dependent neural reactive reorganization.

Consistent with the above framework, research evidence has repeatedly shown that interhemispheric SMC connectivity is positively associated with motor performance—in healthy individuals (Yin et al., 2025; Hensel et al., 2023), in stroke patients (Zhang, 2025), and in SCI patients (Hou et al., 2016). In the present study, resting-state functional connectivity of bilateral sensorimotor cortex (SMC-rsFC) was significantly positively correlated with ASIA sensory and motor scores. These findings further confirm the robust association between SMC-rsFC and sensorimotor function and suggest that reduced interhemispheric SMC connectivity may serve as a candidate biomarker for impaired sensorimotor function in SCI. Future work using dynamic connectivity analyses and longitudinal designs is needed to test the causal predictions of the lateralized compensation hypothesis.

The connectivity of L-PFC ~ R-M1 was weakened, and this change occurs only in HbR rather than HbO2, possibly due to a decrease in local oxygen metabolism without accompanying changes in cerebral blood flow. HbR is sensitive to the reduction in oxygen metabolism caused by impaired neural function, whereas HbO_2_ is mainly influenced by global blood flow regulation. Therefore, in pathological states where neurovascular coupling is impaired, HbR can reflect metabolic changes that HbO_2_ cannot capture (Tam, 2014). This explanation needs to be verified in the future using electrophysiology or metabolic imaging. A further explanation of this change is: The PFC plays a critical role in motor control, including sustaining attention, integrating visual and proprioceptive information, monitoring movement, and participating in planning, coordination, and decision-making processes (Chen X. et al., 2025). From the perspective of motor learning, the PFC is involved in sequential memory storage (Cui et al., 2025) and may facilitate motor skill acquisition by modulating dopaminergic neurons in M1 (Ishikuro et al., 2023). As a core region for motor execution, M1 not only controls motor output but is also closely involved in integrating sensory inputs such as proprioceptive and tactile information. Through precise muscle control and postural adjustments, M1 contributes substantially to postural maintenance and balance recovery. Previous studies have reported that enhanced effective connectivity between the PFC and M1 is associated with improvements in balance performance (Chen X. et al., 2025), motor function (Guo et al., 2022), and the execution of complex directional movements (Liu et al., 2024). Accordingly, the PFC–M1 pathway is considered a critical neural circuit underlying complex motor and balance functions. In the present study, PFC–M1 rsFC did not show a significant correlation with ASIA motor scores. This finding may be related to the fact that the motor tasks assessed by the ASIA motor scale primarily reflect relatively simple voluntary movements rather than complex motor behaviors.

It is also important to note that individuals with SCI may experience various complications in addition to motor impairment, including pain, autonomic dysfunction, spasticity, and depression. Approximately half of individuals with SCI experience neuropathic pain, and long-term functional impairment and persistent pain may adversely affect mental health (Sritharan et al., 2025). For spinal cord injury patients who have never used or have actively discontinued neuropathic pain medications and antidepressants, there is still a high risk of disease due to ongoing endogenous pathological processes and inherent psychosocial factors, even without pharmacological intervention (Ramos and Cruz, 2023). Recent evidence suggests that increased resting-state functional connectivity between the PFC and the motor cortex may be associated with susceptibility to depression (Elfaki et al., 2025). One potential mechanism may involve psychomotor retardation, whereby affective disturbances contribute to slowed or reduced motor activity. Notably, in patients whose depressive symptoms improved following treatment, rsFC between the PFC and motor cortex was found to decrease (Dunlop et al., 2023), suggesting that PFC– motor cortex connectivity may also play a role in mood regulation. In addition, in non-invasive transcranial stimulation treatments used to alleviate neuropathic pain, M1 and PFC are usually chosen as therapeutic targets to reduce pain, suggesting that there may be functional connectivity between M1 and PFC (Zhai et al., 2025). A study on patients with fibromyalgia reported that PFC-M1 connectivity was high regardless of pain severity, providing strong evidence that increased PFC-M1 connectivity may be a biomarker for descending neuropathic pain (De Oliveira Franco et al., 2022).

Taken together, PFC–M1 connectivity appears to be associated not only with complex motor performance but also with mood disorders and neuropathic pain, suggesting that it may represent a common neural pathway underlying these interrelated processes. Therefore, the reduced PFC–M1 connectivity observed in the present study may, on the one hand, be related to impaired complex motor performance in individuals with SCI. On the other hand, it may reflect compensatory functional reorganization associated with spontaneous improvement in depressive symptoms or alleviation of neuropathic pain.

The PFC–M1 common neural pathway hypothesis may partially explain the positive associations observed among depression, disease severity and duration (Wang et al., 2022), motor dysfunction (Benincá et al., 2024), and pain. In the present study, Correlation analysis revealed a significant positive correlation between L-PFC and R-M1 connectivity strength and disease duration, providing additional empirical support for the PFC–M1 common pathway hypothesis. Future repeated-measures studies are needed to validate this shared neural pathway hypothesis.

Quantifiable connectivity based on fNIRS has the potential to be translated into clinically relevant biomarkers. The strong correlation between interhemispheric SMC connectivity and clinical indicators further reinforces this potential. fNIRS may become a valuable clinical tool: that is, to assist in diagnosing patients’ functional deficits and accurately guiding treatment through quantifiable and visualizable biomarkers.

Certain limitations should be acknowledged. First, due to the limited penetration depth of fNIRS, deep structures such as the insula and hippocampus were not captured, so the study was restricted to the cerebral cortex. Second, the cross-sectional design cannot infer causal relationships between disease progression and changes in brain networks. Third, patients were not stratified according to severity of injury or clinical subtype, which may limit the precision of the study results. Finally, due to the limited size of the clinical cohort, the multivariate adjustment analysis in this study was at risk of overfitting and had limited statistical power. Future studies should include patient stratification, utilize multimodal neuroimaging methods (for example, combining fNIRS with fMRI or EEG), and adopt longitudinal designs to examine the sustained changes in clinical indicators and neurobiological markers after interventions. A larger sample size would further enhance the robustness and generalizability of the findings.

## 5 Conclusion

The weakening of interhemispheric SMC connectivity is highly correlated with SCI sensorimotor dysfunction, suggesting it may serve as a potential biomarker for sensorimotor function. Reduced connectivity of PFC-M1 may reflect neural remodeling along common pathways of complex motor function, depression, and neuropathic pain, but this is only a preliminary hypothesis. These quantifiable connectivity features provide potential new targets for monitoring disease progression and evaluating therapeutic interventions, but they need to be further validated in future longitudinal and multimodal studies.

## Data Availability

The original contributions presented in the study are included in the article/Supplementary material, further inquiries can be directed to the corresponding author/s.
